# An Exploratory Study of Cantonese Learning Strategies Amongst Non-Chinese English-Speaking Ethnic Minority University Students in Hong Kong

**DOI:** 10.3389/fpsyg.2022.910603

**Published:** 2022-06-02

**Authors:** Jack Pun, Qianwen Joyce Yu, Tom Keannu Sicuan, Michael Angelo G. Macaraeg, Joe Marc Pineda Cia

**Affiliations:** Department of English, City University of Hong Kong, Kowloon, Hong Kong SAR, China

**Keywords:** ethnic minorities, Cantonese, language acquisition, Hong Kong, learning strategies

## Abstract

This study investigates the strategies for learning Cantonese that are adopted by non-Chinese English-speaking ethnic minority (EM) university students in Hong Kong. The aim is to identify the challenges these students face in applying their strategies to learn Cantonese and to explore their learning experiences when implementing them. Drawing on questionnaire surveys and semi-structured interviews with 30 EM students at a university in Hong Kong, this study identifies these learners’ strategies, elicits their views on the use of these strategies and examines their learning experiences. The findings suggest that EM students are “medium strategy users,” with social strategies being their most commonly used types of learning strategies, followed by compensation and metacognitive strategies. The more proficient Cantonese users tend to use metacognitive strategies that promote planning and are goal-oriented. Taken together, this study sheds light on the complex interplay of sociocultural variables in shaping EM university students’ Cantonese learning experience in Hong Kong. It also highlights the importance of analysing EM students’ linguistic repertoire and the local language ecology in understanding Cantonese learning in a multilingual context like Hong Kong.

## Introduction

As an international city, Hong Kong has a wide array of immigrants from various backgrounds. With ethnic Chinese being the dominant group in Hong Kong, non-Chinese groups (e.g., Filipino, Pakistani, Indian, Nepalese) may try to assimilate into the local culture by adapting and integrating with the majority community. Previous studies demonstrate that non-Chinese groups find Cantonese difficult to master due to its distinctive tone system and its lack of a standardised romanisation system (Li and Richards, 1995; Sachs and Li, 2007).

A sizable gap in Cantonese language proficiency exists between most ethnic minority (EM) students and local Chinese students, even though Chinese is included in the syllabus for non-Chinese speaking students. According to the Census and Statistics Department of Hong Kong (The Census and Statistics Department of Hong Kong, 2016), 52,129 EM students were attending full-time courses at educational institutions in Hong Kong. Considering their different levels of Chinese proficiency, in 2014, the government implemented the General Certificate of Secondary Education (GCSE) in Chinese Language as an alternative curriculum for EM students while retaining the Hong Kong Diploma for Secondary Education in Chinese Language for native Chinese speaking students. The GCSE curriculum covers language skills only up to the primary 3 knowledge standard, and Wong (2016) asserted that the GCSE is insufficient for enabling functional use of the Chinese language. As the GCSE involves much simpler and less demanding tests for non-Chinese speaking students, this programme offers fewer opportunities for EM students to develop and use their own strategies for learning Cantonese.

To date, little research has been conducted in Hong Kong on the language strategies used by EM students. Research in Hong Kong has mainly focussed on the strategies used by Hong Kong Chinese students to learn English or Putonghua (Leung and Hui, 2011). To fill this gap in research, this study aims to identify the sociolinguistic phenomena that influence the language learning strategies that EM university students use to learn Cantonese.

### Ethnic Minority Students in Hong Kong

According to the Census and Statistics Department of Hong Kong (3), 14,054 EM students aged 15 and over and 38,075 EM students under the age of 15 were enrolled in full-time courses at educational institutions in Hong Kong. Designated schools received academic support to strengthen the learning of these EM students in the traditional Chinese curriculum (i.e., Cantonese and Putonghua). The major issue faced by EM students was the limited choice among study programmes, as only four designated public schools were providing curriculum exclusively for EM students (Ku et al., 2005; Kennedy, 2006). It is essential to investigate the efficacy of education policy for teaching EM students to identify the necessary improvements. It is also necessary to explore the types of support that these students may need to learn Cantonese and to evaluate the support systems available, in addition to existing curricula.

### Ethnic Minority Students’ Language Learning Challenges

As the dominant language, Cantonese is widely used by ethnic Chinese in Hong Kong. According to 2016 Population By-Census conducted by the Census and Statistics Department, 88.9% of the total population reported using Cantonese as their usual language (The Census and Statistics Department of Hong Kong, 2016). The popularity of Cantonese is almost impervious to the political change in 1997, when Hong Kong was restored to China (Chan, 2014). While it is an official language, English is generally used in government documents, business sectors, and tertiary education, but not in daily conversation, with only 4.3% of the total population using English as their usual language (The Census and Statistics Department of Hong Kong, 2016). The percentage of residents in Hong Kong using Mandarin as their usual language remained stable at 0.9–1.9% (The Census and Statistics Department of Hong Kong, 2016).

Among EM students aged 5 and over, 45.6% speak English at home and 30.3% speak Cantonese at home (The Census and Statistics Department of Hong Kong, 2016). The lack of opportunity to practise at home poses considerable challenges for EM students’ seeking to learn Cantonese. It is therefore inherently problematic to assume that EM students born in Hong Kong must be fluent in Chinese (Burkholder, 2013). In this situation, school education programmes play a major role in enabling EM students to acquire the Cantonese language. To address this need, the Education Bureau is focussed on enhancing the Cantonese proficiency of EM students by adapting curricula, paedagogies and evaluations to the students’ individual differences and on stimulating their motivation to learn.

The difficulty of learning Cantonese may prevent EM students from integrating into the community and into professional life in Hong Kong (Burkholder, 2013). Various studies have shown that EM students in Hong Kong have difficulty adapting to the local environment (Loper, 2004; Hua et al., 2020). They face limited educational choices and opportunities, limited support for learning Chinese, difficulty attending local public schools and a lack of interaction with Chinese students.

### Language Learning Strategies

Learning strategies can be defined as the “specific actions taken by the learner to make learning easier, faster, more enjoyable, more directed, or effective and able to adapt to new situations” (Oxford, 1990). The two main types of strategies are direct and indirect strategies.

Direct strategies are methods for mentally processing a new language. The three main types of direct strategies are memory, cognition and compensation strategies. Memory strategies involve grouping and using imagery, both of which can help students store and retrieve new information. Cognitive strategies enable learners to understand and produce a new language through various means, such as summarising or reasoning deductively. Compensation strategies allow learners to use the language in situations that involve large knowledge gaps, for example, guessing or using synonyms.

Indirect strategies support and manage language learning without directly using the target language. The three main types of indirect strategies are metacognitive, affective and social strategies. Metacognitive strategies let learners control their own cognition, allowing them to coordinate their learning processes by centring, arranging, planning and evaluating. Affective strategies help learners to better regulate their emotions, motivations, and attitudes. Social strategies help students to learn by interacting with others.

These various strategies can also be categorised according to their mode of operation. Cognitive strategies work with information that enables enhanced learning. Metacognitive strategies involve planning, monitoring or evaluating activities. Social/affective strategies require interaction with others and involve ideational control over affect (O’Malley and Chamot, 1990).

### Research Questions

In Hong Kong, studies of language learning have mainly focussed on the learning strategies that Hong Kong Chinese students adopt to learn English or Putonghua (Leung and Hui, 2011). Less is known about the strategies that EM students use to learn Cantonese. In addition to their challenges in adapting to the sociocultural context during their studies, EM university students also have to fit in with needs of the multilingual environment in Hong Kong. Notably, university students retain their individual background and academic requirements related to individual high school learning experience. Therefore, this study investigated how EM students use Cantonese in tertiary education and what learning strategies they apply to learn Cantonese.

Specifically, this study sought to answer the following research questions:
(1) What strategies do EM students use to learn Cantonese?
(2) What challenges do they face in learning this language?
(3) What are their coping strategies?
(4) What kind of learning experiences do they have when studying Cantonese?

## Materials and Methods

### Research Design

This study adopted a mixed mode approach, combining both quantitative and qualitative components. The first part of the study was a survey, using a quantitative questionnaire to identify the learning strategies used by EM students. With the help of course instructors, the questionnaire was distributed online to the participating students. The questions listed in the survey were randomised since the order of question presentation was likely to influence the participants’ ratings. The second part collected qualitative information through an interview with a selected sample of 10 participants. These participants were selected on the basis of their language proficiency levels indicated by the grades in GCSE Chinese language. Each interview lasted for approximately 45 min. During the interviews, participants described their views and preferences regarding learning strategies and their own learning experiences. The study was approved by the CLASS human research ethics committee of the City University of Hong Kong.

### Participants

Thirty EM university students were randomly recruited, using a stratified sampling technique. The participants were considered eligible if (1) they had completed their secondary school education in Hong Kong (i.e., Grades 7–12); (2) they were currently studying in Hong Kong universities; (3) their first or common language outside of university was English; (4) they had either received a passing grade in GCSE Chinese language (Grade E or above) or had mastered an immediate level of spoken Cantonese; and (5) their parents did not speak Cantonese at home. Written consent was obtained from all participants prior to their participation.

### Instruments

The Strategies Inventory of Language Learning (SILL) questionnaire was adopted to identify the learning strategies used by the participants (Oxford, 1990). This questionnaire includes six categories of strategies, namely memory, cognitive, compensation, metacognitive, affective, and social strategies (see Appendix). The SILL questionnaire has a reliability of 0.93 to 0.98, depending on whether it is translated into another language or into the participant’s own language (Green and Oxford, 1995). In this study, Cronbach’s alpha for the SILL was 0.956, demonstrating the high reliability of this scale for the target sample population. The survey was used to gain a general overview of the experiences of EM students when learning Cantonese in Hong Kong schools. Some of the survey items include the following:

(1) I think of relationships between what I already know and new things I learn in the second language (SL).
(10) I say or write new SL words several times.
(28) I try to guess what the other person will say next in the SL.
(32) I pay attention when someone is speaking the SL.
(40) I encourage myself to speak the SL, even when I am afraid of making a mistake.

To supplement the quantitative data, a qualitative interview was conducted in English with a small sample of 10 participants, as a way to elicit their experiences in using learning strategies for studying Cantonese. The interview protocol was adapted from Leung and Hui’s (2011) study, which involved interviewing students about their attitudes toward learning Mandarin and the strategies they used when learning a new language. Some of the interview questions are listed as follows:

(1) How often do you speak Cantonese?
(2) What learning strategies do you find most useful?
(3) What are your motives for learning Cantonese?

### Data Analysis

The quantitative measures of the variables were managed, described and analysed with SPSS 22.0. This process produced a statistical analysis of the participants and their experiences. The quantitative data were computed for each sub-category score by averaging the students’ ratings of the corresponding survey items.

All interview data were transcribed and translated into English. The researchers conducted a thematic analysis to identify the participants’ views on learning Cantonese and the strategies they used. The research team performed a final double-check of the translations and transcriptions against the original recordings.

## Results

### Strategies Adopted by Ethnic Minority Students

Table 1 presents a description and ranking of the strategies reported by the participants. The mean value of the six categories of learning strategies reported was 3.01, indicating that on average the participants used these strategies to a moderate degree.

**TABLE 1:** Mean values, standard deviations and ranking of the reported strategies (by category).

Language learning Strategies	*M* (*SD*)	Rank order
Social strategies	3.5 (0.88)	1
Compensation	3.35 (0.63)	2
Metacognitive	3.02 (1.0)	3
Cognitive	2.93 (0.78)	4
Memory	2.7 (0.76)	5
Affective	2.53 (0.80)	6

A mean between 3.5 and 5.0 for an SILL item was considered to reflect the participants’ high use of that strategy. A mean of 2.5 to 3.4 reflected medium use, and a mean of 1.0 to 2.4 reflected low use (Corp, 2020). As shown in Table 1, “social strategies” (*M* = 3.5, *SD* = 0.88) were the most frequently used, followed by “compensation” (*M* = 3.35, *SD* = 0.63) and “metacognitive” (*M* = 3.02, *SD* = 1.0) strategies. The least commonly used types of strategies were “affective strategies” (*M* = 2.53, *SD* = 0.80). All of the strategies except “social strategies” fell into the mean range between 2.4 and 3.4, so they could be considered “medium use” language learning strategies. Evidently, the participating EM students used these strategies only occasionally (Oxford, 1990). They reported using social strategies most frequently, as shown by the mean value (3.5 > 3.4) for these types of strategies.

### Frequency of Strategy Use

The most commonly used strategies are identified in Table 2. Among the top 10 strategies, nine had a mean value between 3.5 and 5.0. According to Oxford (1990), this range of values is considered to indicate “high usage.” The most frequently chosen strategies were types of social, compensation and metacognitive strategies.

**TABLE 2:** Strategies most frequently used by students (in descending order).

Item	Strategy	Category	*M* (*SD*)
24.	To understand unfamiliar Cantonese words, I make guesses.	(Compensation)	4.34 (0.76)
32.	I pay attention when someone is speaking Cantonese	(Metacognitive)	4.08 (0.91)
25.	When I can’t think of a word during a conversation in Cantonese, I use gestures.	(Compensation)	3.92 (1.1)
11.	I try to talk like native Cantonese speakers.	(Cognitive)	3.86 (1.2)
45.	If I do not understand something in Cantonese, I ask the other person to slow down or say it again.	(Social strategies)	3.86 (1.1)
50.	I try to learn about the culture of Cantonese speakers.	(Social strategies)	3.78 (1.2)
48.	I ask for help from Cantonese speakers.	(Social strategies)	3.69 (1.2)
29.	If I can’t think of a Cantonese word, I use a word or phrase that means the same thing.	(Compensation)	3.67 (1.0)
31.	I notice my Cantonese mistakes and use that information to help me improve.	(Metacognitive)	3.64 (1.2)
47.	I practise the sounds of Cantonese.	(Cognitive)	3.5 (1.0)
46.	I ask Cantonese speakers to correct me when I talk.	(Social strategies)	3.44 (1.4)

Among the top 10 strategies, four were types of social strategies. These were item 45 (*If I do not understand something in Cantonese, I ask the other person to slow down or say it again*) (*M* = 3.86), item 50 (*I try to learn about the culture of Cantonese speakers*) (*M* = 3.78), item 48 (*I ask for help from Cantonese speakers*) (*M* = 3.69) and item 46 (*I ask Cantonese speakers to correct me when I talk*) (*M* = 3.44). Three of the strategies listed in the top 10 were types of compensation strategies. These included item 24 (*To understand unfamiliar Cantonese words, I make guesses*) (*M* = 4.33), item 25 (*When I can’t think of a word during a conversation in Cantonese, I use gestures*) (*M* = 3.92) and item 29 (*If I can’t think of a Cantonese word, I use a word or phrase that means the same thing*) (*M* = 3.67). Two of the top 10 strategies were types of metacognitive strategies. These included item 32 (*I pay attention when someone is speaking Cantonese*) (*M* = 4.08) and item 31 (*I notice my Cantonese mistakes and use that information to help me improve*) (*M* = 3.64).

The most frequently used strategy was a type of compensation strategy, namely item 24 (*To understand unfamiliar Cantonese words, I make guesses*) (*M* = 4.33). The second most frequently used strategy was item 32 (*I pay attention when someone is speaking Cantonese*) (*M* = 4.08), which was a type of metacognitive strategy. Items 24 and 32 had a mean above 4. This set of findings was consistent with the mean scores of the top three strategies reported in Table 1: social strategies (*M* = 3.5), compensation strategies (*M* = 3.35) and metacognitive strategies (*M* = 3.02).

To supplement the questionnaire results, we conducted in-depth interviews with 10 participants to elicit their observations and experiences with learning Cantonese. These interviews were conducted in a hybrid manner with either face-to-face or online Zoom meetings. Ten of the 30 survey participants were selected based on their language proficiency levels indicated by the grades in GCSE Chinese language (see Table 3 for demographic details) and invited for a follow-up interview.

**TABLE 3:** Demographic Details of the Interviewees.

Interviewees (pseudonyms)	Ethnicity	Language backgrounds	Cantonese proficiency
Nadia	Afghani/Pakistani/Filipino	First language: English Second language: Cantonese Third language: Pashto	High
Jack	Pakistani	First language: Pashto Second language: English Third language: Cantonese	High
Nipurna	Nepalese	First language: Nepali Second language: English Third language: Cantonese	High
Noshela	Pakistani	First language: Urdu Second language: English Third language: Cantonese	High
Rajib	Filipino-Pakistani	First language: English Second language: Cantonese	Middle
Armin	Filipino	First language: English Second language: Tagalong Third language: Cantonese	Low
Jose	Filipino	First language: Tagalog Second language: English Third language: Cantonese	Low
Zack	Pakistani	First language: English Second language: Cantonese	High
Anthony	Filipino	First language: English Second language: Cantonese	Low
Kuldeep	Indian	First language: Punjabi Second language: English Third language: Cantonese	Low

First, they were asked about their language backgrounds and learning environments. Four of them had high-level Cantonese proficiency and another four had low proficiency. Half of the interviewees spoke Cantonese as their third language, and four spoke it as their SL. Most of the interviewees were of Filipino descent and the others were of Pakistani, Nepali, or Indian origins. All interviewees were born in Hong Kong, grew up with English as their common language and had acquired some understanding of Cantonese over the course of their upbringing.

Next, they were asked about the challenges and difficulties they faced in learning Cantonese. They were also asked to describe their coping strategies to deal with these challenges.

### Overall Effective Strategies

Nine of the interviewees mentioned that socialising with other Cantonese speakers enabled them to deal with learning challenges. Moreover, four mentioned that this was their most effective strategy and five said that this strategy worked for them. The interviewees strongly believed that immersing themselves in the local culture could help to enhance their Cantonese proficiency. They reported that talking to locals helped them learn new words and phrases and to get correction when they made mistakes. The interviewees also mentioned that conversing in Cantonese with their peers helped them gain confidence in their language skills.

***Nipurna:** So every time, whenever I talked to them, I would learn new words, or a new phrase, like a lot of different things that I didn’t learn in high school*.
***Nadia:** And sometimes if I [made mistakes in Cantonese], they would correct me right away. And they gave me more realistic or accurate words than those you get learning online*.
***Jack:** The most effective way to learn Cantonese would be to put yourself in that environment, to surround yourself with Cantonese speaking people and to force yourself to read more, write more, listen more and speak more*.
***Armin:** [Talking with Cantonese speakers] made me more confident about my speaking ability*.

Social strategies were the most popular type of strategy, as the most commonly used strategies involved socialising. For instance, item 24 (*To understand unfamiliar Cantonese words, I make guesses*) (*M* = 3.86) and item 46 (*I ask Cantonese speakers to correct me when I talk*) (*M* = 3.44) could both be applied in group dialog. These were high usage strategies, as they were rated above the mean of 3.4. Six interviewees also mentioned that they enhanced their Cantonese proficiency by consuming Chinese media such as television shows or Chinese texts. This set of findings was related to one of the top social strategies, namely item 50 (*I try to learn about the culture of Cantonese speakers*) (*M* = 3.78). Therefore, social strategies were clearly the most commonly used types of strategies.

When asked to identify which strategies worked best for them, two of the interviewees said that they incorporated guessing into their learning efforts. Both Jack and Nipurna made this claim, and they were both high proficiency speakers. This observation is consistent with the survey finding that one of the most frequently used strategies was item 24: *To understand unfamiliar Cantonese words, I make guesses* (*M* = 4.34). As Cantonese is a tonal language, all of the interviewees affirmed the importance of practising to speak like a local Cantonese and of rehearsing the pronunciation of Cantonese words. Hence, item 11 (*M* = 3.86) and item 47 (*M* = 3.5) were among the top strategies used.

***Jack:** I also make guesses sometimes* … *for example, in a reading passage, uh, my teacher taught me. when you’re reading and you encounter a word that you don’t know, you just try to guess the meaning of the word based on what the sentence is trying to say, or what the context is exactly. Sometimes I was right and sometimes not. Then I would analyse why I was right or why I was wrong, and this* … *really helped me a lot*.
***Nipurna:** Um, the most effective [strategy] for me is guessing. Guessing what they meant. Yeah, because if you know what 99% of the sentence means, it’s very easy to guess the 1% you did not understand …, and I think that’s pretty effective. So I continue using that*.

### Language Learning Challenges

Among the six types of strategies listed, the participants encountered the greatest difficulties with the auditory, verbal and emotional aspects of the social and affective categories of learning strategies. They reported having the most difficulty using social strategies. Items 45 (*M* = 3.86), 46 (*M* = 3.44) and 47 (*M* = 3.04) involved the greatest challenges in terms of attaining proficiency in spoken Cantonese. The next most challenging set of tasks involved using affective strategies. The participants’ ratings for items 42 and 39 (*M* = 3.14 and 3.00, respectively) indicated their emotional struggles when trying to use Cantonese. These two items of affective strategies received an average score of 3.0, which indicated that the participants viewed these strategies as their greatest learning challenges.

Compared with the difficulties that the participants reported when performing cognitive, metacognitive and compensation tasks, they reported even greater difficulties understanding Cantonese. They gave an average score of 2.6 for items involving comprehension-related challenges, such as items 18 (s*kimming a passage*), 26 (*making up new words*), 27 (*reading without looking up every new word*), 36 (*reading as much as possible in Cantonese*), and item 35 (*looking for opportunities for verbal practice*). The challenges they reported regarding these categories were not as great as those involving social and affective strategies. However, these challenges were also important, as this study sought to identify all of the participants’ learning challenges in all of the categories of analysis.

The top 10 challenges presented in Table 2 were consistent with those reported by the 10 interviewees. Although these interviewees preferred socialising in Cantonese as their top learning strategy, six of them reported difficulties with social interaction in Cantonese, such as issues with pronunciation due to the complexity of tonal differences and the limited vocabulary of the less experienced students.

***Noshela:** I also tried to talk to my friends in Chinese so that I could improve and they could correct my Chinese*.

Similar to the survey participants who reported various reading difficulties, four of the interviewees also reported reading comprehension problems. They described using various coping strategies such as memorisation and constant exposure to Chinese vocabulary as a way to improve their knowledge and understanding of more Cantonese words.

Furthermore, due to their inexperience with socialising in Cantonese, five of the interviewees reported feeling anxious and unconfident when communicating with local speakers in Cantonese. These responses confirmed the survey findings on the participants’ difficulties using affective strategies due to being nervous or afraid when studying or using Cantonese.

***Anthony:** It’s not that I didn’t want to learn Cantonese. I think it’s mostly because I was too scared to interact in Cantonese …*.

### Least Effective Language Strategies

The participants found the learning strategies in the memory and cognitive categories to be the least helpful. Specifically, they found these strategies unfruitful for enabling competence in Cantonese. In the memory category, items 5 (*M* = 2.14), 6 (*M* = 1.64) and 7 (*M* = 2.19) were perceived as inadequate learning or coping strategies, and they received low ratings. Likewise, the participants tended to avoid cognitive strategy methods such as items 15 (*M* = 2.17), 16 (*M* = 1.61) and 17 (*M* = 1.64).

Only one participant found memorisation to be an effective strategy for learning Cantonese.

***Jack:** I really could connect the sounds of Cantonese words or like pictures to help me remember these words. Also by making a mental picture of a situation where the word might be used … [Memorisation] was a great coping strategy for me*.

Although a few interviewees used memorisation methods such as repetition when learning Cantonese, three of the interviewees claimed that this was ineffective due to the tonal variations and other difficulties of speaking Cantonese. Regarding the use of cognitive strategies, several interviewees felt that engaging with Chinese media, for example, by reading Chinese texts or watching Chinese movies or television shows, was an unattractive learning strategy. Although some interviewees engaged in this practice, it was not as popular as socialising. Two of the 10 interviewees claimed that engaging with Chinese media was ineffective because of its difficulty.

***Anthony:** I have tried consuming some (Chinese) media, but as my friends speak English, it doesn’t really give me an incentive to continue because I have no one to talk to about these*.

In addition, most of the survey participants viewed leisure strategies (such as rewarding themselves or writing down their feelings in a learning diary) to be ineffective. They felt that these strategies were impractical for improving their proficiency in Cantonese.

### Learning Experiences

Three of the interviewees provided detailed observations that encapsulated not only the recurring patterns found among the 10 interviewees but also described their unique formal learning environment. These interviewees emphasised the lack of readily available resources to aid in learning Cantonese and the lack of uniformity in resources across different schools that offer various modes of instruction. These responses highlighted the issues and difficulties that EM students face in learning Cantonese.

These three interviewees, namely Noshela, Rajib and Kuldeep, had different levels of Cantonese proficiency, but the experiences they reported were representative of most of the survey participants and the interviewees. This consistency in responses from the participants with different levels of proficiency in both the survey and interview phases of the research indicated common themes and patterns of experience.

These three interviewees were of South Asian descent, with Rajib having mixed lineage from Southeast Asia. All of them were born and raised in Hong Kong and had gone through the local school curriculum from kindergarten to secondary school. Noshela had a high level of proficiency in Cantonese, and her first language was Urdu. Rajib had middle-level proficiency in Cantonese, and his first language was English. Kuldeep had low- to middle-level proficiency in Cantonese, and his first language was Hindi. All three of these first languages use either alphabets, abugidas, or abjads (alphabetic writing systems) that differ greatly from the logographic characters used in Cantonese. An important point of their discussion was the stage at which they were exposed to Cantonese. Noshela had enrolled in a Chinese Medium of Instruction (CMI) school at an early age, but Rajib and Kuldeep were educated mainly in English Medium of Instruction (EMI) schools. The importance of the educational background and the language of instruction is discussed in the next sections. The experiences of these interviewees illustrate a common pattern in which out-of-school learning regimens/strategies are needed to support students, or to compensate for the shortcomings of their formal classroom instruction.

#### High-Frequency Cantonese-Using Participants

Noshela was a Pakistani born and raised in Hong Kong, who had been learning Cantonese since she was 3 years old. She spoke the language fluently and used it daily alongside English. She described her learning environment as difficult, as she was enrolled in CMI schools from kindergarten until secondary school, but her mother tongue was Urdu. In addition to describing her formal education, she noted that she had started to study Cantonese on her own when she was around 9. She tried to talk to her peers in Cantonese, seeking to improve and correct her errors. She mentioned that most of her education in Cantonese happened in school, but her most effective learning strategies were engaging in social interaction and cultural immersion.

Noshela mentioned that she was not keen on learning Cantonese during her formal education from kindergarten to primary school. She found Cantonese difficult, regardless of the number of years she had spent learning it.

***Noshela:** Until I was in primary 4 or 5, I was not really keen on learning Cantonese because I found it very difficult to pick up. Although I had been learning it already for like 5 or 6 years, it was still difficult for me to pick up or even recognise new words*.

A change in her perception motivated her to learn more actively when she began studying at a CMI school, which meant that all of her classes were taught in Cantonese. At this point, she no longer saw Cantonese as an option but rather as a necessity to continue her education. She started by reading Chinese texts to expand her vocabulary and familiarise herself with the characters. She noted that her greatest challenges in learning Cantonese were mastering the pronunciation and vocabulary (reading and writing), along with the tonal distinctions and the arbitrariness of the sounds and the characters, which were totally unlike the letters used in English. She explained that her Chinese language curriculum was designed for the EM system in EMI schools. This curriculum is a simplified programme that does not equip students with the necessary tools to hone their skills in Cantonese and does not provide the kind of cultural immersion and social interaction required to attain proficiency.

***Noshela:** So the thing is, there’s this curriculum in schools in which they try to have a simplified programme for EM students, so that they can learn Chinese easily because the curriculum is simplified and old. [Also] you are grouped with other EM students, which means you’re having class with other EM students, but not Chinese students. I think that’s the risk(iest) strategy for me …*.

*If you’re just asking EM students to learn with their EM counterparts, [then] all of us don’t know Cantonese*… *what’s the point of learning a language when you’re not even immersed or exposed to the culture*… *if you’re immersed with other EM students*,… *the result is that most of them speak in English*… *the teacher doesn’t even touch the difficult [words in Cantonese]*.

Noshela cited her CMI background as one of the biggest contributions to her Cantonese proficiency. However, she still needed to supplement what she learnt in class by seeking her own additional resources. She practised writing and expressing her thoughts in Cantonese with the help of her dictionary. She also practised speaking with native Cantonese peers to overcome her learning difficulties.

#### Middle-Frequency Cantonese-Using Participants

Rajib was a Pakistani-British student who was born in the Philippines and raised in Hong Kong. His first language was English, which he continued to use daily. He started learning Cantonese at a local kindergarten and in EMI primary and secondary schools. During his kindergarten years, he had no substantial practice in Cantonese aside from the help he received from his parents.

***Rajib:** In kindergarten, there wasn’t really a set curriculum when it came to learning Chinese. At that time, it was mostly through, like, trying to interact with my classmates. But from primary school and secondary school, we did have formal instruction in Cantonese, you know, Chinese language classes*.

His formal education in Cantonese started in primary school. Then in his secondary school, Cantonese classes were segregated between local students for Chinese literature studies and EM students, who had a very simplified Chinese curriculum. They learnt phrases similar to what people learn from tourist guide books, rather than the types of speech used in daily conversation. Rajib felt that his greatest challenge was the mismatch between students who could not communicate in Cantonese and their teachers who had difficulty communicating in English.

***Rajib:** The main challenge is that the teachers assigned to teach Chinese language to EM students aren’t necessarily equipped to properly connect and communicate with EM students. Teachers don’t necessarily know how to communicate effectively in English so there was like a lot of butting of heads when it comes to being able to communicate effectively. So that does not help us when it comes to trying to get our language skills up to par*.

Rajib believed that this intercultural (mis) communication contributed to his lack of understanding in classroom sessions. He pointed out that speaking was less difficult for him, and he tried to approach his local classmates to practise communication in Cantonese. He found, however, that for EM students like himself, writing [and reading] was more difficult. He explained that he was able to read materials in class, but because of the repetitive structures and intertwined vocabulary, he was not able to apply his skills outside of the classroom. He overcame these challenges by actively seeking free out-of-school resources, such as tutorial sessions at his local community centre. These sessions, however, were not always accessible, and at some point they ended completely due to lack of funding.

***Rajib:** The vocabulary tends to be interrelated so you are able to parse it a lot easier. But when you step out of the classroom and try to apply these skills, it doesn’t necessarily work*.

*When it comes to EM students, most of the exposure they had was only inside the classroom. So when I was in secondary school, I tried to solve that problem by, you know, having after-school tutoring sessions at my local community centre, because they used to run free Cantonese classes. Once they stopped funding these classes, I ran into the same problems again*.

Rajib next discussed his preference for social interactions as a learning strategy. He found this strategy to be more helpful than memorisation because it was more organic and it enabled better retention. He also mentioned that although there was adequate support for students like him, most of the education funding went to CMI schools, which typically did not admit EM students. As a result, most EM students had to seek additional resources on their own.

***Rajib:** I did a bit of research on this [and found] that the effectiveness of these types of schemes is limited because in the first place*… *a lot of CMI schools that don’t accept that many EM students don’t actually benefit much from this funding. Even if they do receive funding, it is not enough to be able to train teachers to speak English effectively, to, you know, communicate with their EM students. And for these students, it’s a shame because even if they are put in these situations–even if they receive support, they do not necessarily benefit from it*.

#### Middle- to Low-Frequency Cantonese-Using Participants

Kuldeep was an Indian national who was born and raised in Hong Kong. His first language was Punjabi and he spoke English for daily interactions outside his home. He started his Cantonese learning experience from kindergarten and learnt basic vocabulary until secondary school. He did not talk much of his primary school experience, but he emphasised that the secondary school curriculum in Cantonese hindered his ability to use Cantonese for daily interactions. He mentioned that the students were taught enough to pass and do well in their GCSE exams, but this did not translate into language proficiency outside of the school setting. He explained that it was not his school’s formal education that helped him speak Cantonese fluently, but rather his habit of spending afternoons after school watching Cantonese-dubbed television programmes, which he would discuss with his local peers at school to practise his speaking and listening.

***Kuldeep:** Most of the Cantonese I learnt was from coming home and watching TV; I would come home and watch TV in Chinese. That’s how I learnt how to speak Chinese*.

*I would come home and watch anime. watch a Chinese dubbed anime on TV each day. So that’s how I learnt how to speak fluently in Chinese. And*… *because I watched anime in Chinese, I could, uh, speak to my, uh, Chinese speaking friends about the anime. I had like local friends from, uh, tutoring centres. And so we would talk about some anime we watched and that’s how I developed my speaking skills in Chinese*.

Kuldeep’s greatest challenges were developing his reading and writing skills, as the words he learnt in class did not equip him with the vocabulary and grammar repertoire to create sentences to express his ideas and opinions outside of school. He mentioned the lack of real-life application of the language. He had difficulty matching his development in speaking and listening skills with his reading and writing skills.

***Kuldeep:** [The school] prepared us to take the exam. The exam being the GCSE, they taught us so. We could do well on the exam, but maybe that does not reflect what we would need on a daily basis. For example, if I were to apply for a job interview and the interview would be conducted in Chinese, they did not teach these skills. During my secondary school, they just taught us how to take the exam. So that’s one critique I have*.

*I had a worse time reading and writing, um, than speaking and listening, I had an easier time grasping the knowledge [when trying to speak]. I had more practice and more [opportunities] to practise my listening skills and speaking skills, but for reading and writing, I did not need to express myself by writing in Chinese. I did not need to read Chinese text every day. So they definitely did not develop the necessary skills to read in Cantonese or write in Cantonese*.

Kuldeep overcame these difficulties with his mother’s support by hiring a Cantonese tutor. The tutor helped him practise what he learnt in class and provided further help to develop his Cantonese proficiency.

***Kuldeep:** I did have tutoring classes and my mum hired a tutor for my exams, um, for my GCSE exams. He taught me to write and read, uh, Chinese characters*.

Kuldeep noted that he tried to hone his reading and writing skills through reading Cantonese comics, but the words used were too complex for him to understand anything.

***Kuldeep:** I tried to approach [the problem] by reading more Chinese literature. Not literature per se. I came across this Chinese comic, “*
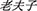
***”** and read it. I would try to understand what the characters were saying in the comic and that’s how I tried to tackle the issue. But unfortunately it was not successful because the words in the comics were too complex, and I could not understand anything. So I still struggle with reading and writing Chinese*.

He further explained that support was available for EM students in Hong Kong, but it was not as readily available as he would have liked. He referred to his experience as a typical example. His family hired a tutor and some of his peers were studying Cantonese *via* tutoring classes offered by the police department, but these classes were not endorsed by his school. Thus, he discovered that EM students generally had to find resources for training themselves.

### Common Themes and Patterns

After collating the data from the 30 questionnaire participants and the 10 interviewees, a number of common learning experiences, challenges, preferred strategies, common themes and patterns emerged. The challenges faced by these learners of Cantonese appeared to have three main sources: the inadequate curriculum, the lack of exposure to conversations with locals and the inadequate availability of out-of-school resources/materials for learning Cantonese. Although the participants’ preferred strategies for learning varied to some extent, they generally found that social strategies were the most helpful, with less preference for compensation and/or metacognitive strategies. What stood out in the three analysed cases was the common themes and patterns that these students shared with the other interviewees. The following subsections report how the remaining seven interviewees viewed and dealt with these themes and patterns.

#### Curriculum Design

The interviewees repeatedly mentioned that Cantonese class curricula generally lacked “real-life” and “practical” content applicable for daily use outside of the classroom. The interviewees claimed that the curricula for EM students were designed solely to prepare students for exams and did not equip them with the speaking, reading and writing skills they would need to fully/partially assimilate into the local/dominant Chinese speaking culture in Hong Kong.

***Armin:** Like in school I never really learnt anything. And just because I feel like I’m not learning anything, I’m not listening, you know? Yeah. And I can’t read, or I can’t write because the school*,… *like I said, they train you to memorise words, but not really use the language. So when I see [a word] in the street, I know what it means, but when I see it in a sentence, I’m just like, I don’t, I don’t know. Because they teach you basic words or. things like that*.

***Nipurna:** First, as I was in a segregated school, um, a lot of us thought Chinese was not important. So we thought Chinese was just a course we needed to pass in high school. And in high school, they don’t teach you Chinese according to the formula. When you’re in Forms 1 to 6, you only learn Chinese up to primary 3 standard. So we always have this picture in our mind that Chinese is easy, because it was very easy. Our exams would just be like, they would give us a Chinese passage. And then the question would be in English, and then we just have to find an English answer from the Chinese passage. So like, they present Chinese as a very easy course, but it’s not easy. And we weren’t improving*.

***Nadia:** [The curriculum] has different options [that students] can choose for the GCSE exams. They can choose IGCSE, SAT or GC, A-levels. But for the workplace, again, it goes back to the start. I feel like all that progress goes back to the start. [In the workplace] they just focus on your Cantonese fluency and they judge you based on that rather than your skills. They feel more comfortable with someone who can speak Cantonese rather than someone who can’t*.

The interviewees briefly mentioned the initiatives they took to overcome their difficulties with the curriculum. A conclusive analysis of the data collected, along with statistical data, is presented in the following sections and discussed in terms of (a) strategy preferences, (b) strategy effectiveness, and (c) literature review and consolidation of the findings on social strategies.

#### Common Challenges

When asked about their challenges in learning Cantonese, many of the interviewees categorised their problems in relation to three specific elements of the language: reading, writing and speaking. Each of these three elements involved particular challenges. In learning to write and read, the interviewees reported difficulty memorising the different Chinese characters and learning the various tones that accompany each character. For speaking and listening, they described their lack of opportunity to practise with local Cantonese speakers in their formal education setting, because as EM students, they were enrolled in mixed schools with EM and native Chinese students segregated into separate Cantonese classes.

##### Memorisation of Chinese Characters and Tones

The majority of our interviewees’ mother tongues are written with alphabetic systems such as Latin alphabets, abjads or abugidas, to name but a few. Few of the interviewees’ first languages were tonal languages, although they did feature varied inflexions, expressions and/or intonations. Some of the difficulties encountered by the interviewees when learning Cantonese were caused by their lack of exposure to the cultural context of the Chinese characters, which made it difficult for them to gain both an in-depth understanding and sensitivity to the distinctions between the tones used in the language.

***Anthony:** [The greatest challenge] would be the sheer volume of vocabulary and the pronunciation … The vocabulary, it was hard for me when I was a kid to remember the characters and how to say them, because my languages are English and Tagalog, I didn’t have experience with the tones. So it was really hard for me to remember them or pronounce them*.
***Nadia:** Yeah, nine tones. It’s really hard. Yeah. Like, my teacher had to make a separate class for me just to get the pronunciation right. And my writing, I think it’s okay. But speaking is a little, just the sound is weird*.
***Zack:** Definitely memorising the characters. You know, the Chinese characters, they’re not easy to remember. So definitely memorising the characters. Also, it’s just very easy for me to forget these Chinese characters*.

##### Lack of Practice Speaking Cantonese With Locals

As many of the interviewees mentioned, EM students are generally segregated from local students, so they have little opportunity to practise speaking Cantonese in class or at school in general. Many of the interviewees said that they had hoped to have some integration with local Cantonese speaking students to facilitate real-time listening and speaking practice. These EM students found that speaking/practicing Cantonese with their fellow EM students helped to boost their confidence, but it did not help them to deal with their problems of limited vocabulary or incorrect tones. In some cases, the interviewees had no one in their school to speak Cantonese with.

***Jose:** The biggest challenge is not having people to talk to, to practise with you, you know, because you and your friends would probably have different levels when speaking Cantonese. So it would be really difficult if you didn’t have any Chinese friends. So that’s the biggest challenge*.
***Nipurna:** The Chinese section and the English section were divided. So if you wanted to learn Chinese, the only source was from Chinese classes … but there weren’t many opportunities for [EM students] to actually interact with [local students in the Chinese section]. So we rarely talked to [students in] the Chinese section and we rarely had any Chinese friends at school*.
***Nadia:** [Local students] automatically speak English with me. Even when I try to speak Cantonese with them, they shift back to English because they want to improve their own English. So I haven’t really had a chance to use Cantonese in the past 4 years. I think I’ve forgotten a lot [of Cantonese]. It’s gotten worse*.

#### Commonly Preferred Strategies and the Availability or Quality of External Resources

The interviewees expressed a common feeling about the availability and quality of out-of-school resources for learning Cantonese. Many of them said that there was ample government funding for government-sponsored initiatives to teach Cantonese through cultural immersion programmes, such as participating in Chinese musical productions or attending extracurricular tutoring classes outside of schools. However, such programmes were often unavailable or not endorsed by their schools. The schools themselves could have done a better job of endorsing these external resources. Due to these gaps in the availability of extracurricular programmes, the majority of the interviewees developed their own preferred learning strategies to compensate for the shortcomings of formal training in their respective schools. Most of the interviewees found that they preferred social strategies for learning. These strategies included cultural immersion activities such as participating in Chinese-language musicals, talking in Cantonese with locals or their peers or learning Cantonese through music and film.

***Jose:** The thing that would help all students learn is to just talk in Chinese when you’re not in class, because that’s where we really learn. One can try to make Chinese learning interesting for you, so you could maybe check out Chinese songs or shows, or watch Chinese films with English subtitles. Just try to learn that way*.
***Jack:** I also talked a lot with my [Cantonese/local classmates] and so I could hear [the language] more …. Eventually you just get used to it and it becomes a form of habit. This coping strategy improved my listening and speaking at the same time because I had to speak more and receive answers. So my fluency also improved*.
***Nipurna:** In terms of self-study, I would speak Chinese every day because I would talk to my neighbours, the security guard, or those who live in my building. I would use Chinese outside more than I used it in school. And I think I learnt more from outside than I did from school*.
***Armin:** I joined this Chinese musical performance and it really helped me practise my Cantonese more. I was with friends, so it was not necessarily them teaching me, but more practicing*.

## Discussion

### Strategy Preferences

Overall, social strategies were the highest rated category among the learning methods (*M* = 3.5), followed by compensation (*M* = 3.35) and metacognitive strategies (*M* = 3.02). These results are slightly different from those found by other researchers on SL learning, such as Leung and Hui (2011), who analysed Hong Kong Putonghua learners, or Bremner (Oxford and Burry-Stock, 1995), who examined learners of English as a second language (ESL) in Hong Kong. The top categories of learning strategies found in these two studies were compensation and metacognitive strategies, rather than social strategies. These discrepancies in results probably reflect the fact that there are abundant opportunities to use social strategies in areas where the Cantonese-speaking community is dominant, unlike the situation of English learners, who have comparatively fewer opportunities to practise outside of classroom settings. Another factor in these divergent results could be the relatively low level of Cantonese language proficiency among this study’s participants. Their overall proficiency score was 2.7, and 80% of the participants had only completed the GCSE curriculum. Therefore, they had a greater need to use item 45 (*If I do not understand something in Cantonese, I ask the other person to slow down or say it again*) when interacting with locals. They also had a greater need to rely on other Cantonese speakers when trying to communicate in Cantonese. Therefore, they relied on item 48 (*I ask for help from Cantonese speakers*). These findings are consistent with those of Lunt (2000) and Bremner (2016), who examined the learning strategies of immigrant ESL learners in Australia and the results of the study by Wharton (2000), in which learners in Singapore showed a strong preference for social strategies, as they “needed to use these strategies to survive.” Li and Richards (1995), who conducted a survey of people learning Cantonese as an additional language, found that most respondents needed Cantonese mainly for transactional purposes. The top-ranked situations were, in descending order, (a) buying things in the marketplace, (b) taking a taxi, (c) asking for directions, and (d) ordering food. These findings are similar to our interviewees’ observations. When asked if Cantonese fluency was essential for them, all of the interviewees agreed that it was, and they mentioned that it was necessary for daily transactions and communications.

The results showed that compensation and metacognitive strategies were the second and third most popular learning methods, as has been found in most other studies. One factor affecting this pattern of results could be the study participants’ lack of Cantonese proficiency. According to Oxford (1990), compensation strategies are generally adopted to make up for an inadequate grammar and vocabulary repertoire. Thus, non-fluent learners typically have a greater need to use guessing (item 24: *To understand unfamiliar Cantonese words, I make guesses*, *M* = 4.34) and to use gestures (item 25: *When I can’t think of a word during a conversation in Cantonese, I use gestures*, *M* = 3.92). Learners typically use these strategies to compensate for their lack of vocabulary and their inability to communicate and form sentences clearly. Guessing and making gestures helps them deliver and convey their messages the best they can, and thus these methods were the study participants’ second most commonly used strategies. Metacognitive strategies were the third most commonly used, but these initiatives for planning methods of learning were not necessarily created by the participants themselves. Item 34 (*I plan my schedule so I will have enough time to study Cantonese*, *M* = 1.92) was one of the lowest ranked metacognitive strategies and, in general, the metacognitive methods were considered low-use strategies.

### Strategy Effectiveness

When asked which Cantonese learning strategies they found most effective, the interviewees reported their greatest successes in using compensation, metacognitive and social strategies. These responses coincided with the survey findings on the ranking and preference of learning strategies. The more proficient Cantonese speakers used more metacognitive strategies than the less proficient students, which is consistent with the findings of Leung and Hui (2011). The participants with a better command of the language tended to take more initiative to find new ways to improve their Cantonese. For instance, the participants who gave a score of 5 (strongly agree) to item 35 (*I look for people I can talk to in Cantonese*) (*M* = 2.69) had an average self-rated Cantonese proficiency score of 4. However, the 11 participants (30.6%) who gave a score of 4 (agree) to item 37 (*I have clear goals to improve my Cantonese skills*) (*M* = 2.53) had an average self-rated Cantonese proficiency score of 3.73. Only one participant who gave a score of 5 to item 37 had a Cantonese proficiency self-rating score of 5. According to O’Malley and Chamot (1990); Bedell and Oxford (1996), and Vandergrift (1999) the use of metacognitive strategies increases proportionally with increasing levels of proficiency. The researchers in this study found a difference in the learning strategies used by beginners and by intermediate learners. The intermediate learners used more metacognitive strategies. Studies have shown that metacognitive strategies help learners to learn SL more proficiently and effectively (Vandergrift, 1999; Anderson, 2002). Metacognitive strategies are essential in helping learners to identify what they are trying to accomplish, to consider what strategies they are using, to assess their progress consciously and to consider alternative strategies (Wenden, 1986). Therefore, the more proficient Cantonese users in this study tended to use metacognitive strategies that promoted goal-oriented planning.

### Learning Challenges

The participants reported that their greatest challenges in learning Cantonese were related to their attempts to use social strategies. The means of their scores on the difficulty of these strategies were above 3.0. Wenden (1986) and Li and Chuk (2015) found similar results regarding EM students’ levels of competency in Chinese. They showed that although EM speakers were surrounded by native Cantonese speakers and had no lack of exposure to written Chinese, few of them managed to overcome the “Chinese literacy gap” and the “Cantonese deficiency syndrome.” Moreover, Li and Chuk (2015) observed that although their EM subjects were commonly experienced language learners, their bilingual or multilingual repertoires were of little help to them when learning written Chinese and spoken Cantonese. The main reason why our participants encountered pronunciation and comprehension difficulties was that Cantonese is a tonal language. Recent studies have determined that Cantonese learners find the sounds of the six tones difficult to discern, as the “same word” can have different meanings due to the slight differences in tone (Sachs and Li, 2007; Wong, 2016). Furthermore, as EM students’ homeland languages and English are (usually) not tonal languages, these students lack sensitivity to the fine tonal differences in Cantonese words, and they frequently repeat their errors when learning Cantonese (Defrancis, 2002; Li and Chuk, 2015). Therefore, the difficulty of hearing the differences between very similar sounding Cantonese words was the greatest challenge for this study’s participants.

Many participants reported having emotional difficulties when learning Cantonese. In the affective strategy category, two items received a mean score above 3.0 regarding the levels of anxiety experienced when trying to use Cantonese. In addition to their difficulties with pronunciation and comprehension, the participants were often embarrassed by their mistakes. As Noshela reported,

*It’s very embarrassing to say so, but like, I used to cry whenever I had to answer questions in Chinese class, because I didn’t understand Chinese. I didn’t really understand Chinese. And I was really scared of giving the wrong answer or even saying the wrong words. So whenever I had to answer a question, I would just say “um. Uh” and then I would just, you know, cry*.

The studies by Wenden (1986); Li and Chuk (2015), and Wong (2016) obtained similar results, indicating that EM learners commonly feel embarrassed by their mispronunciation and other mistakes when trying to speak Cantonese. These researchers showed that EM students felt demotivated because of the laughter and uncooperative or unfriendly reactions they received when mispronouncing Cantonese syllables. As a result, they felt discouraged from learning Cantonese and their emotional struggles became barriers to learning the language. These findings are consistent with the challenges that the participants in this study reported regarding their use of affective learning strategies.

In terms of literacy development, the participants reported several issues regarding their knowledge of Cantonese writing. For example, they had difficulty understanding Cantonese words that were new to them or understanding Cantonese content. These challenges are expected, as written Chinese uses a non-alphabetic symbols. In comparison, English provides phonetic cues for pronouncing each specific word, and the alphabetic writing system enhances phonemic awareness regarding the sequence of letters and the pronunciation patterns. These pronunciation cues are not possible with standard written Chinese. As Chinese written forms are largely divorced from pronunciation, it follows that regular exposure to and practice of writing Chinese characters is necessary for learners to enhance their skills in written Chinese and spoken Cantonese.

Jack, one of the interviewees, reported that one of his coping strategies to improve his Cantonese was to learn new words to expand his knowledge and vocabulary:

*[My friends] told [me] to write down the Cantonese words that I don’t know. So I learn a new word every day, maybe learn 5 to 10 new words if I can. In the beginning, I couldn’t learn 5 to 10 words. I could only learn 2 to 3, and then slowly I could learn 5 to 10 new words. … So this really increased the vocabulary range of my Cantonese*.

The difficulties encountered by EM learners in learning spoken Cantonese lead to additional problems in their acquisition of reading and writing skills, as speech is commonly recognised as a factor contributing to the acquisition of literacy or reading skills in any language (Education Bureau, 2008). In any writing system, alphabetic or otherwise, most of the words rely on phonetic (as opposed to visual) cues for effective decoding (Defrancis, 2002). As a result, the experiences of EM learners in reading or writing Chinese are complicated by their gaps in understanding spoken Cantonese. These differences in the Chinese written system and in spoken Cantonese make the language difficult to remember, difficult to practise and easy to forget (Education Bureau, 2008). More opportunities of exposure to Cantonese and written Chinese are therefore of great significance for EM learners in Hong Kong, which generally functions in trilingualism. The findings resonate with Erbaugh (2002) and Li et al. (2020), who claim that such opportunities can empower EM learners in the establishment of an elaborated social network with Cantonese-speaking peers.

As with all research, the findings are presented alongside their limitations. This study is a small-scale study of participants’ perceptions of their Cantonese learning experience. The results are based on a single university and thus limited in generalisability. Future research may seek to investigate the impact of mixture of languages, and participant variables such as socioeconomic status, and linguistic and educational background.

## Conclusion

This study has shown that EM students are typically “medium strategy users.” Among the six categories of learning strategies, the participants most commonly relied on social strategies, with compensation and metacognitive strategies being their second and third most favoured types of strategies. These findings differ from those of previous studies involving Chinese ESL learners, but they are similar to the results of studies involving immigrant SL learners. The participants in this study also reported that they commonly used compensation strategies, although over-reliance on these strategies could be problematic. Taguchi (Li et al., 2020) suggested that as learners progress in their language journey, they should avoid using compensation strategies that might negatively affect their vocal communication. Instead, the author recommended using other methods such as metacognitive strategies, which can help learners review their language development and learning tasks.

It is important to note the challenges that EM students face in their Cantonese learning experiences. To understand why and how they have developed their own preferences and strategies for use outside of the traditional teacher/classroom–student setting, it is helpful to identify and analyse the common patterns and themes of barriers to learning, and then to gain a solid understanding of learning strategies, based on data on the experiences of many learners. Throughout this study, the identification of the participants’ preferred learning strategies was matched with background investigations of the challenges involved. Most of the participants reported a lack of *empathy* in the traditional language-learning setting. Empathy, as a resource in foreign language acquisition, involves a development of interpersonal *understanding* between instructor and learner–in this case understanding between Cantonese teachers (in both EMI and CMI schools) and their EM students. The participants described a lack of empathetic resources, for example, a lack of focus on learning Cantonese that is relevant to real-life/practical skills such as finding a job or handling conversations in the workplace. The participants described the need for better opportunities to develop their learning skills through practices such as speaking in casual (out-of-textbook) conversations. Such conversations can enable learners to master the cadences and nuances of spoken language much better than learning through textbooks. Other issues reported by the EM learners included the distinct differences in writing systems, phonology, semiotics and morphology between the learners’ primary languages and Cantonese. The results of this study showed that empathetic resources, or the lack thereof, have a major effect on learners’ ability to find their own preferred learning strategies.

The EM students in this study had their greatest success using social, metacognitive and compensation strategies, as these strategies helped them deal with their greatest challenges. The greatest problems for these EM Cantonese students were pronunciation problems and issues with self-esteem and self-perception regarding their language proficiency. The differences between the Chinese written system and the spoken vernacular Cantonese made mastering Cantonese an arduous task for these EM students. To learn the language successfully, they needed constant exposure and familiarity with the language. Clearly, certain coping strategies help EM students to learn Cantonese more effectively and proficiently. Nonetheless, learning to use Cantonese beyond the classroom remains a major challenge. Findings of the present study may contribute to broadening the research scope of Cantonese learning in the multilingual environment of Hong Kong. A focus on EM university students in Hong Kong can not only provide insights into what challenges and opportunities they encounter in Cantonese learning and how students strive to improve their learning experience, but it can also generate useful information for language learners who might be going through similar changes in other contexts. The study points to the dynamic nature of Cantonese learning process and unpacks the various influencing factors within the EM communities of Cantonese learning. Such knowledge can further provide useful implications for instructors and policymakers about how to support EM students in coping with various obstacles in language learning.

## Data Availability Statement

The raw data supporting the conclusions of this article are available from the corresponding author on reasonable request.

## Ethics Statement

The studies involving human participants were reviewed and approved by the CLASS Human Research Ethics Committee of the City University of Hong Kong. The patients/participants provided their written informed consent to participate in this study.

## Author Contributions

All authors listed have made a substantial, direct, and intellectual contribution to the work, and approved it for publication.

## Conflict of Interest

The authors declare that the research was conducted in the absence of any commercial or financial relationships that could be construed as a potential conflict of interest.

## Publisher’s Note

All claims expressed in this article are solely those of the authors and do not necessarily represent those of their affiliated organizations, or those of the publisher, the editors and the reviewers. Any product that may be evaluated in this article, or claim that may be made by its manufacturer, is not guaranteed or endorsed by the publisher.
