# Supplementary material for: An Exploratory Study of Cantonese Learning Strategies Amongst Non-Chinese English-Speaking Ethnic Minority University Students in Hong Kong
Source: Front Psychol. 2022 Jun 2;13:910603. doi: 10.3389/fpsyg.2022.910603 (PMC9200990; doi:10.3389/fpsyg.2022.910603)
Supplement: Supplementary file 1 [file Data_Sheet_1.docx]

Appendix 1: Questionnaire

**Cantonese Learning Strategies amongst the non-Chinese English Speakers in the City University of Hong Kong**

We would like to invite you to participate in this research project by completing the following questionnaire. This project, conducted by the Department of English of the City University of Hong Kong, aims to investigate the strategies for learning Cantonese that are adopted by non-Chinese English-speaking ethnic minority university students in Hong Kong. Your participation is entirely voluntary and your responses will be treated with extreme confidentiality. Thank you in advance for your cooperation.

**Part I Demographic information**

Please provide the following information by filling in the blanks.

1. Your age: ______

2. Your ethnicity: ______

3. Your first language: ______

4. Your second language: ______

5. What language do you normally use to interact or communicate? ______

6. What is your preferred language for communication? ______

**Part II Language Learning (Memory)**

Please answer in terms of how well the statement describes you. Do not answer how you think you should be, or what other people do. There are no right or wrong answers to these statements. (1= Strongly Disagree; 2= Disagree; 3= Neutral; 4= Agree; 5= Strongly Agree.)

| 7. I think of relationships between what I already know and new things I learn in Cantonese | 1 | 2 | 3 | 4 | 5 |
| --- | --- | --- | --- | --- | --- |
| 8. I use new Cantonese words in a sentence so I can remember them. | 1 | 2 | 3 | 4 | 5 |
| 9. I connect the sound of a Cantonese word and an image or picture of the word to help me remember the word. | 1 | 2 | 3 | 4 | 5 |
| 10. I remember a new Cantonese word by making a mental picture of a situation in which the word might be used. | 1 | 2 | 3 | 4 | 5 |
| 11. I use rhymes to remember new Cantonese words. | 1 | 2 | 3 | 4 | 5 |
| 12. I use flashcards to remember new Cantonese words. | 1 | 2 | 3 | 4 | 5 |
| 13. I physically act out new Cantonese words. | 1 | 2 | 3 | 4 | 5 |
| 14. I review Cantonese lessons often. | 1 | 2 | 3 | 4 | 5 |
| 15. I remember new Cantonese words or phrases by remembering their location on the page, on the board, or on a street sign. | 1 | 2 | 3 | 4 | 5 |
| 16. I say or write new Cantonese words several times. | 1 | 2 | 3 | 4 | 5 |
| 17. I try to talk like native Cantonese speakers. | 1 | 2 | 3 | 4 | 5 |
| 18. I practice the sounds of Cantonese. | 1 | 2 | 3 | 4 | 5 |
| 19. I use the Cantonese words I know in different ways. | 1 | 2 | 3 | 4 | 5 |
| 20. I start conversations in Cantonese. | 1 | 2 | 3 | 4 | 5 |
| 21. I watch SL language TV shows spoken in SL or go to movies spoken in Cantonese. | 1 | 2 | 3 | 4 | 5 |
| 22. I read for pleasure in Cantonese. | 1 | 2 | 3 | 4 | 5 |
| 23. I write notes, messages, letters, or reports in Cantonese. | 1 | 2 | 3 | 4 | 5 |
| 24. I first skim a Cantonese passage (read over the passage quickly) then go back and read carefully. | 1 | 2 | 3 | 4 | 5 |
| 25. I look for words in my own language that are similar to new words in Cantonese. | 1 | 2 | 3 | 4 | 5 |
| 26. I try to find patterns in Cantonese. | 1 | 2 | 3 | 4 | 5 |
| 27. I find the meaning of a Cantonese word by dividing it into parts that I understand. | 1 | 2 | 3 | 4 | 5 |
| 28. I try not to translate word for word. | 1 | 2 | 3 | 4 | 5 |
| 29. I make summaries of information that I hear or read in Cantonese. | 1 | 2 | 3 | 4 | 5 |
| 30. To understand unfamiliar SL words, I make guesses. | 1 | 2 | 3 | 4 | 5 |
| 31. When I can't think of a word during a conversation in the SL, I use gestures. | 1 | 2 | 3 | 4 | 5 |
| 32. I make up new words if I do not know the right ones in the SL. | 1 | 2 | 3 | 4 | 5 |
| 33. I read SL without looking up every new word. | 1 | 2 | 3 | 4 | 5 |
| 34. I try to guess what the other person will say next in the SL. | 1 | 2 | 3 | 4 | 5 |
| 35. If I can't think of an SL word, I use a word or phrase that means the same thing. | 1 | 2 | 3 | 4 | 5 |
| 36. I try to find as many ways as I can to use my SL. | 1 | 2 | 3 | 4 | 5 |
| 37. I notice my SL mistakes and use that information to help me do better. | 1 | 2 | 3 | 4 | 5 |
| 38. I pay attention when someone is speaking SL. | 1 | 2 | 3 | 4 | 5 |
| 39. I try to find out how to be a better learner of SL. | 1 | 2 | 3 | 4 | 5 |
| 40. I plan my schedule so I will have enough time to study SL. | 1 | 2 | 3 | 4 | 5 |
| 41. I look for people I can talk to in SL. | 1 | 2 | 3 | 4 | 5 |
| 42. I look for opportunities to read as much as possible in SL. | 1 | 2 | 3 | 4 | 5 |
| 43. I have clear goals for improving my SL skills. | 1 | 2 | 3 | 4 | 5 |
| 44. I think about my progress in learning SL. | 1 | 2 | 3 | 4 | 5 |
| 45. I try to relax whenever I feel afraid of using Cantonese. | 1 | 2 | 3 | 4 | 5 |
| 46. I encourage myself to speak Cantonese even when I am afraid of making a mistake. | 1 | 2 | 3 | 4 | 5 |
| 47. I give myself a reward or treat when I do well in Cantonese. | 1 | 2 | 3 | 4 | 5 |
| 48. I notice if I am tense or nervous when I am studying or using Cantonese. | 1 | 2 | 3 | 4 | 5 |
| 49. I write down my feelings in a language learning dairy. | 1 | 2 | 3 | 4 | 5 |
| 50. I talk to someone else about how I feel when I am learning Cantonese. | 1 | 2 | 3 | 4 | 5 |
| 51. If I do not understand something in Cantonese, I ask the other person to slow down or say it again. | 1 | 2 | 3 | 4 | 5 |
| 52. I ask Cantonese speakers to correct me when I talk. | 1 | 2 | 3 | 4 | 5 |
| 53. I practice Cantonese with other students. | 1 | 2 | 3 | 4 | 5 |
| 54. I ask for help from Cantonese speakers. | 1 | 2 | 3 | 4 | 5 |
| 55. I ask questions in Cantonese. | 1 | 2 | 3 | 4 | 5 |
| 56. I try to learn about the culture of Cantonese speakers. | 1 | 2 | 3 | 4 | 5 |

Appendix 2: Interview guide

1. Can you please briefly introduce yourself and your linguistic background?

2. When did you start learning Cantonese? How long have you been learning Cantonese?

3. Can you please describe your learning environment? Did you learn Cantonese at school? From formal instruction? Or from informal instruction, by a close friend who is a native speaker? Or a tutor? or by yourself?

4. From experience, what were the challenges when learning Cantonese?

5. According to your experience, how did you address these challenges? Can you please describe each challenge and each coping strategy you used in the past?

6. Which coping strategies did you find the most effective to address the challenges? Can you please elaborate on each strategy?

7. According to your experience, which was the most effective way to learn Cantonese? / What were the strategies that work for you and ones that don’t? Can you please elaborate on each strategy?

8. Do you think there has been adequate support towards Cantonese learning strategies for non-Cantonese speakers in Hong Kong according to your experience? E.g. in schools, as a curriculum. If yes, why? If not, why?

9. Can you share your journey of learning Cantonese as an ethnic minority student in Hong Kong? What was the most challenging part? How did you manage/monitor your study? What impression/evaluation do you give yourself about your Cantonese learning experience?

10. According to your experience, do you think Cantonese fluency is essential to you?
